# Three-Dimensional Micro-CT Assessment of Radiopacity and Intra-Cavity Distribution of Universal Adhesives

**DOI:** 10.3390/ma19143080

**Published:** 2026-07-17

**Authors:** Hidenori Hamba, Takashi Nakano, Shimon Okamura, Takashi Muramatsu

**Affiliations:** Department of Operative Dentistry, Cariology and Pulp Biology, Tokyo Dental College, 2-9-18, Kanda-Misakicho, Chiyoda-ku, Tokyo 101-0061, Japansokamura@tdc.ac.jp (S.O.); tmuramat@tdc.ac.jp (T.M.)

**Keywords:** universal bonding agent, radiopacity, micro-CT, cylindrical cavity, adhesive layer thickness, three-dimensional analysis

## Abstract

The radiopacity of adhesives is important for radiographic diagnosis and micro-computed tomography (micro-CT) evaluation of adhesive restorations. However, most universal adhesives lack sufficient radiopacity. This study examined the radiopacity and intracavity distribution of four universal adhesives—Scotchbond Universal Adhesive (SBU), Scotchbond Universal Plus Adhesive (SBP), Clearfil Universal Bond Quick ER (UBQ), and G-Premio Bond (GPB)—using a cylindrical cavity model. Cavities were prepared in bovine enamel–dentin blocks and scanned using micro-CT at 5.0 μm/voxel after cavity preparation, adhesive application, and resin composite filling (*n* = 3). The adhesive layer region was geometrically defined from sequential datasets and analyzed using actual 16-bit grayscale values. Grayscale values, adhesive layer thickness, and composite-filling-induced volume changes were evaluated by three-dimensional difference analysis. SBP showed a grayscale value equivalent to that of dentin, whereas SBU, UBQ, and GPB were statistically indistinguishable from air. Adhesive thickness increased from the lateral walls to the cavity floor, and reduced-density regions were observed in UBQ and GPB. Therefore, SBP was the only tested universal adhesive with dentin-equivalent radiopacity, enabling reliable micro-CT evaluation and reducing the risk of radiographic misdiagnosis. The proposed grayscale-based approach may support future non-destructive investigations of dental adhesive behavior.

## 1. Introduction

Micro-computed tomography (micro-CT) is widely used for the non-destructive three-dimensional evaluation of dental restorative materials, including adhesive interfaces, gap formation, and polymerization shrinkage in resin composite restorations [1]. This non-destructive approach has recently been extended to the micro-CT evaluation of the internal adaptation of restorations placed using universal adhesives [2]. For accurate micro-CT analysis, the target material must exhibit sufficient radiopacity to be distinguished from adjacent tooth structures and air spaces. When grayscale values are close to those of air or dentin, threshold-based segmentation becomes unreliable, potentially affecting quantitative volume and interface measurements [3].

Although ISO 4049:2019 defines the radiopacity requirements for polymer-based restorative materials [4], no equivalent standard exists for dental adhesive materials. Previous studies have consistently demonstrated the insufficient radiopacity of adhesives. Hotta and Yamamoto reported that 14 of 15 adhesives were less radiopaque than dentin [5], whereas Oztas et al. [6] and de Moraes Porto et al. [7] reported similar findings for contemporary adhesive systems. However, these studies mainly used flat disk specimens, which do not reproduce the three-dimensional distribution of adhesives within an enclosed enamel–dentin cavity.

Insufficient radiopacity of adhesives may also affect clinical radiographic diagnosis. Kursun et al. reported false-positive diagnoses of simulated secondary caries adjacent to bonding-agent-lined restorations [8]. Fröhlich et al. showed that multiple layers of a universal adhesive increased the risk of misidentifying radiolucent zones and recommending unnecessary restoration replacement [9], whereas Pamir et al. associated adhesive pooling at the cavity floor with false-positive replacement diagnoses [10]. Consistent with these observations, a recent study confirmed that lower restorative radiopacity reduces the diagnostic accuracy for secondary caries [11].

In micro-CT studies, Sumitani et al. showed that adhesive layers in a cylindrical cavity model had grayscale values closer to air than to dentin, thereby compromising interface segmentation [3]. Zaharia et al. demonstrated that detectable magnetic nanoparticles enabled three-dimensional visualization and volumetric quantification of dental adhesives using micro-CT [12]. These findings indicate that adhesive detectability is essential for reliable micro-CT-based evaluation.

Universal adhesives are widely used because of their simplified application and versatility [13]; however, their radiopacity and intracavity distribution have not been sufficiently investigated using micro-CT. Scotchbond Universal Plus Adhesive (SBP; Solventum) reportedly achieves radiopacity through a bisphenol A (BPA)-free, HEMA-free radiopaque crosslinking monomer [14,15]. However, independent quantitative evaluations of this property within a cavity model remain limited.

This study aimed to investigate the radiopacity and intracavity distribution of four universal adhesives in a standardized cylindrical cavity model using micro-CT and to evaluate the effect of resin composite filling on adhesive distribution. The null hypothesis was that there would be no significant differences in radiopacity among the tested universal adhesives or between the adhesives and dentin.

## 2. Materials and Methods

### 2.1. Specimen Preparation

Bovine anterior teeth were commercially obtained as food-processing by-products (Yokohama, Kanagawa, Japan) and sectioned into enamel–dentin blocks (4 × 4 × 3 mm) using a precision low-speed saw (Isomet; Buehler, Lake Bluff, IL, USA). A standardized cylindrical cavity (1.5 mm diameter, 2 mm depth) was prepared on the labial surface of each block using a desktop drill and cylindrical diamond bur (FG#202CR; Shofu, Kyoto, Japan). Reference holes were prepared on the lateral sides of each specimen block to serve as fiducial markers for three-dimensional image registration across sequential micro-CT scans.

### 2.2. Adhesive Materials and Application

Four universal adhesives were evaluated (Table 1): Scotchbond Universal Adhesive (SBU; Solventum, Maplewood, MN, USA; Lot 4897805), SBP (Solventum; Lot 01746A), Clearfil Universal Bond Quick ER (UBQ; Kuraray Noritake Dental, Tokyo, Japan; Lot 7K0016), and G-Premio Bond (GPB; GC, Tokyo, Japan; Lot 2001161). Each adhesive was applied by a single operator to the cavity surface in the self-etch mode according to the manufacturer’s instructions, including material-specific air-drying procedures, and light-cured for 10 s using a light-emitting diode (LED) light-curing unit (Pencure 2000; J. Morita, Osaka, Japan) in the standard mode. The cavity was then filled with a flowable resin composite (RC; Clearfil Majesty ES Flow Low; Kuraray Noritake Dental; Lot 620320) and light-cured for 10 s. Three specimens were prepared for each adhesive group (*n* = 3). This sample size is consistent with a previous micro-CT study of dental restorative materials that used a comparable methodology (*n* = 3) [3], and reflects the requirement of the present sequential-scanning protocol to image each specimen under identical exposure and reconstruction conditions at every experimental step.

### 2.3. Micro-CT Scanning

Each specimen was scanned using a micro-CT system (SMX-100CT; Shimadzu, Kyoto, Japan) under the following conditions: tube voltage, 100 kV; tube current, 70 μA; beam filter, 0.2 mm brass; frame averaging, 10; image matrix, 1024 × 1024 pixels; isotropic voxel size, 5.0 μm; and 500–600 slices. Scans were performed at three sequential time points: after cavity preparation, after adhesive application, and after resin composite filling. The reduced specimen size of 4 × 4 × 3 mm enabled high-resolution imaging at 5.0 μm/voxel, yielding 36 scan datasets across the four adhesive groups. The sequential micro-CT scanning protocol was adapted from previous investigations [3,16].

### 2.4. Three-Dimensional Image Analysis

Three-dimensional image analysis was performed using TRI/3D-BON64 software (version 10.0; Ratoc System Engineering Co., Ltd., Tokyo, Japan) with the sequential difference analysis option. The three sequential scan datasets were semi-automatically registered using the reference holes on the lateral sides of each specimen as fiducial markers. After spatial registration, each dataset was binarized using a threshold optimized to segment the cavity space from the surrounding tooth structure. This software has previously been used for the micro-CT-based volumetric evaluation of dental restorative materials [17].

For adhesive layer analysis, the adhesive layer region was geometrically defined from the sequential datasets by subtracting the resin composite region from the cavity space in the post-filling dataset. CT data within this region were extracted and analyzed using actual 16-bit grayscale values without threshold-based binarization of the adhesive. This approach allowed adhesive layer thickness and internal grayscale variations to be evaluated in all four adhesive groups, irrespective of their inherent radiopacity. Mean grayscale values were measured for the adhesive, resin composite, enamel, dentin, and air. Adhesive layer thickness was measured at five positions: the lateral cavity walls at 0.5, 1.0, and 1.5 mm from the cavity entrance; the cavity corner; and the cavity floor. These data were presented descriptively to characterize the three-dimensional distribution patterns.

To evaluate the effect of resin composite filling on adhesive distribution, the total adhesive volume was obtained from the post-adhesive application binarized dataset after morphological void filling. The altered volume was calculated by subtracting the post-filling binarized dataset from the post-adhesive application binarized dataset. The volume change ratio was calculated as the altered volume divided by the total adhesive volume and expressed as a percentage.

Ring-shaped reconstruction artifacts, which occurred around the central X-ray beam axis, were observed in all specimens. Although these artifacts were reduced by image filtering, slight residual artifacts persisted. Therefore, grayscale value measurements and adhesive layer thickness analyses were performed at the cavity walls and floor, away from the artifact region. Observer blinding was not feasible for the SBP group because of its distinguishable radiopacity. All measurements were performed by a single trained operator according to predefined criteria.

### 2.5. Statistical Analysis

Differences in grayscale values among groups were evaluated using one-way analysis of variance (ANOVA) followed by the Games–Howell post hoc test, which does not assume equal variances, because Levene’s test indicated heterogeneity of variances among the groups (*p* = 0.018). Adhesive layer thickness was compared among the four adhesives at each measurement position using one-way analysis of variance. For the SBP group, pre- and post-filling adhesive volumes were compared using a paired t-test. The significance level was set at *p* < 0.05. All statistical analyses were performed using IBM SPSS Statistics version 27 (IBM Corp., Armonk, NY, USA).

## 3. Results

### 3.1. Representative Micro-CT Images

**Figure 1:**
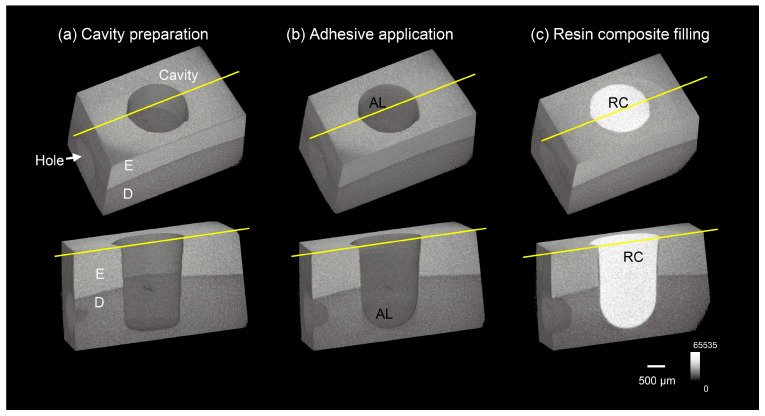
Representative three-dimensional micro-CT images of the SBP group after (**a**) cavity preparation, (**b**) adhesive application, and (**c**) resin composite filling. **Upper row**: surface views; **lower row**: cross-sectional views. The arrow indicates the lateral reference hole used for image registration. The yellow lines indicate the cross-sectional planes. E, enamel; D, dentin; AL, adhesive layer; RC, resin composite; micro-CT, micro-computed tomography; SBP, Scotchbond Universal Plus Adhesive. Ring-shaped reconstruction artifacts were observed at the center of the cylindrical cavity. Scale bar = 500 μm. The grayscale bar indicates the 16-bit grayscale values.

**Figure 2:**
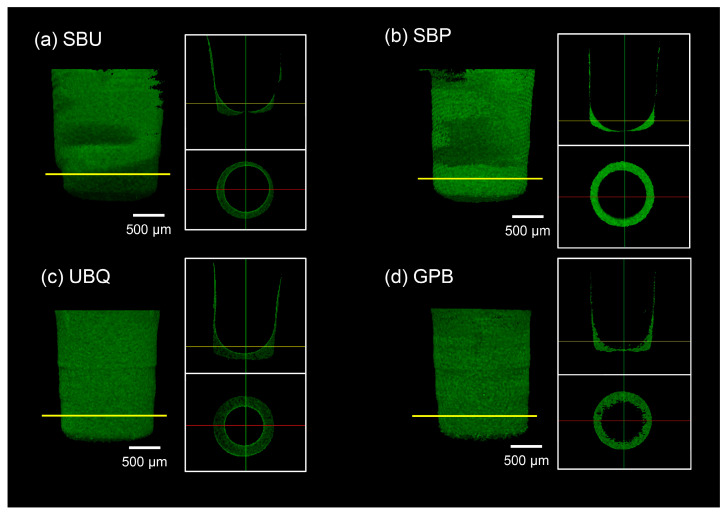
Three-dimensional micro-CT images of the geometrically defined adhesive layer regions. (**a**) SBU, (**b**) SBP, (**c**) UBQ, and (**d**) GPB. For each group, the **left panel** shows a three-dimensional rendering and the **right panel** shows orthogonal cross-sections. The adhesive layer region was geometrically extracted from the sequential micro-CT datasets and displayed using the actual CT grayscale values. In the orthogonal cross-sectional panels, the green and red lines indicate the positions of the intersecting section planes. A ring-shaped distribution along the lateral walls and pooling at the cavity corner and floor were observed in all groups. SBU, Scotchbond Universal Adhesive; SBP, Scotchbond Universal Plus Adhesive; UBQ, Clearfil Universal Bond Quick ER; GPB, G-Premio Bond; micro-CT, micro-computed tomography. Scale bar = 500 μm.

**Figure 3:**
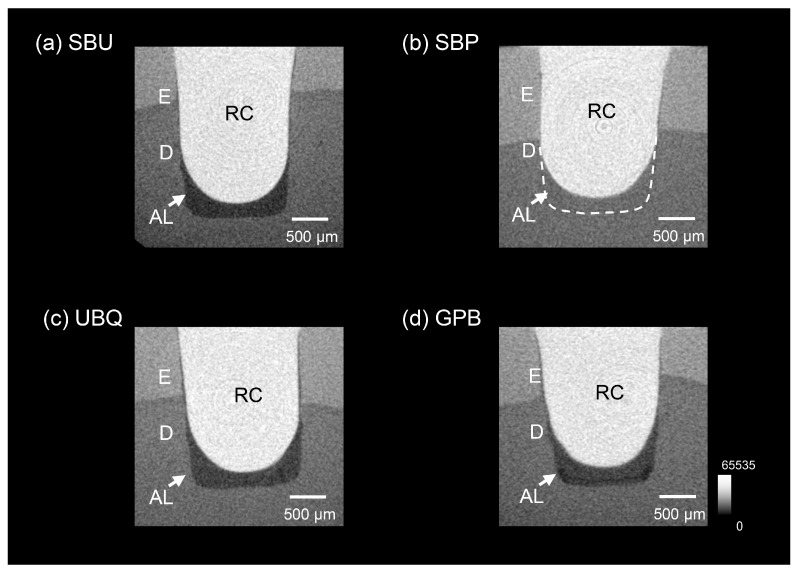
Two-dimensional cross-sectional micro-CT images of the cavity region after resin composite filling: (**a**) SBU, (**b**) SBP, (**c**) UBQ, and (**d**) GPB. The SBP adhesive layer was clearly distinguishable between the tooth substrate and resin composite, as indicated by the dashed line. In SBU and UBQ, the adhesive layer was difficult to identify because of its low radiopacity. The arrowheads indicate reduced-density regions within the GPB adhesive layer. E, enamel; D, dentin; AL, adhesive layer; RC, resin composite; micro-CT, micro-computed tomography; SBU, Scotchbond Universal Adhesive; SBP, Scotchbond Universal Plus Adhesive; UBQ, Clearfil Universal Bond Quick ER; GPB, G-Premio Bond. Scale bar = 500 μm. The grayscale bar indicates the 16-bit grayscale values.

Representative three-dimensional micro-CT images of the SBP group are shown in Figure 1, illustrating the sequential changes after cavity preparation, adhesive application, and resin composite filling. Extracted adhesive layer regions for all four groups are shown in Figure 2, and two-dimensional cross-sectional images after resin composite filling are shown in Figure 3.

The SBP adhesive layer showed clear contrast with the surrounding dentin and cavity space, consistent with its dentin-equivalent radiopacity. In contrast, the SBU and UBQ adhesive layers showed low contrast and were difficult to identify visually. In GPB, reduced-density regions became apparent within the adhesive layer only after resin composite filling, when the radiopaque resin composite provided sufficient contrast. These regions were not discernible in the post-adhesive application images and were not observed in SBU or SBP. Ring-shaped reconstruction artifacts were observed around the central X-ray beam axis in all specimens but did not interfere with the evaluation of the cavity walls.

### 3.2. Grayscale Values and Radiopacity

**Figure 4:**
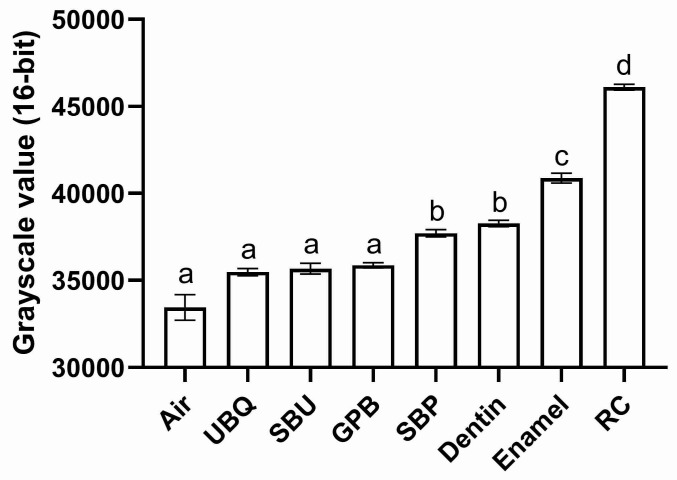
Mean 16-bit grayscale values of the evaluated materials in the cylindrical cavity model (*n* = 3). Error bars indicate standard deviations. Different letters indicate significant differences among groups (one-way ANOVA with Games–Howell post hoc test, *p* < 0.05). SBU, Scotchbond Universal Adhesive; SBP, Scotchbond Universal Plus Adhesive; UBQ, Clearfil Universal Bond Quick ER; GPB, G-Premio Bond; RC, resin composite; ANOVA, analysis of variance.

The mean 16-bit grayscale values are shown in Figure 4 and Table 2. Based on one-way ANOVA followed by the Games–Howell post hoc test, the tested materials were classified into homogeneous subsets, indicated by the letters a–d in Figure 4 and Table 2; materials sharing the same letter did not differ significantly (*p* > 0.05). The values ranged from 33,440 ± 735 for air to 46,103 ± 164 for the resin composite. Among the four adhesives, SBP showed the highest grayscale value (37,716 ± 207), which was significantly higher than that of air and not significantly different from that of dentin (38,279 ± 187), indicating dentin-equivalent radiopacity. In contrast, SBU, UBQ, and GPB showed grayscale values that were statistically indistinguishable from those of air and significantly lower than those of dentin (*p* < 0.05). The resin composite and enamel showed the highest grayscale values and differed significantly from all other materials and from each other.

### 3.3. Adhesive Layer Thickness Distribution

**Figure 5:**
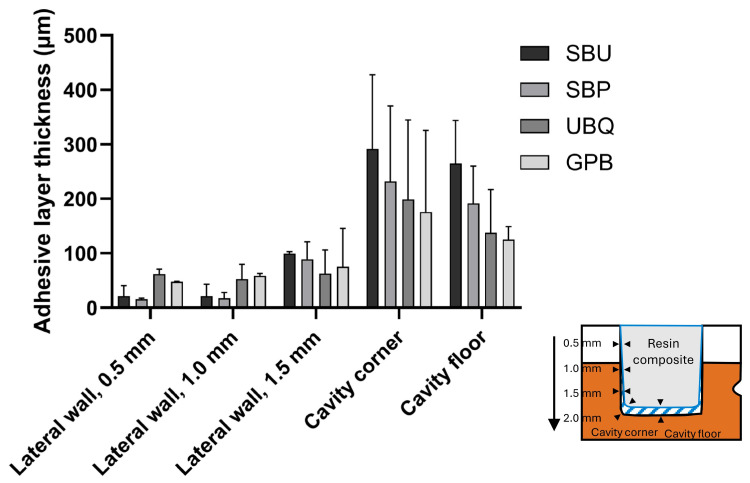
Adhesive layer thickness at each measurement position across the four adhesive groups. Data are shown as mean ± SD (μm, *n* = 3). The measurement positions included the lateral cavity walls at depths of 0.5, 1.0, and 1.5 mm from the cavity entrance, cavity corner, and cavity floor. The inset shows the measurement positions. In the inset, the blue outline indicates the restoration, the gray region indicates the resin composite, the hatched area indicates the adhesive layer, and the arrows indicate the five measurement positions along the cavity wall and floor. SBU, Scotchbond Universal Adhesive; SBP, Scotchbond Universal Plus Adhesive; UBQ, Clearfil Universal Bond Quick ER; GPB, G-Premio Bond; SD, standard deviation.

Adhesive layer thickness at each measurement position is described in Figure 5 and Table 3, as the primary aim was to characterize the intracavity distribution pattern of the adhesive. Across all groups, the adhesive layer was thin at the lateral cavity walls and markedly thicker at the cavity corner and floor. One-way analysis of variance revealed no significant differences in thickness among the four adhesives at any measurement position (*p* > 0.05), indicating that this intracavity distribution pattern was consistent regardless of adhesive type.

At the lateral walls, the mean thickness ranged from 15.0 ± 17.3 μm (SBP) to 68.3 ± 55.1 μm (UBQ) at a depth of 0.5 mm; from 25.0 ± 10.0 μm (SBP) to 71.7 ± 33.3 μm (UBQ) at a depth of 1.0 mm; and from 93.3 ± 32.5 μm (UBQ) to 125.0 ± 25.0 μm (GPB) at a depth of 1.5 mm. At the cavity floor, the mean thickness was 321.0 ± 209.6 μm for SBU, 240.0 ± 142.9 μm for SBP, 194.0 ± 81.6 μm for UBQ, and 142.0 ± 108.4 μm for GPB. The overall mean thickness was 66.1 μm at the lateral walls and 224.2 μm at the cavity floor, representing an approximately 3.4-fold increase from the wall to the floor.

### 3.4. Volume Change in Adhesive Following Resin Composite Filling

**Figure 6:**
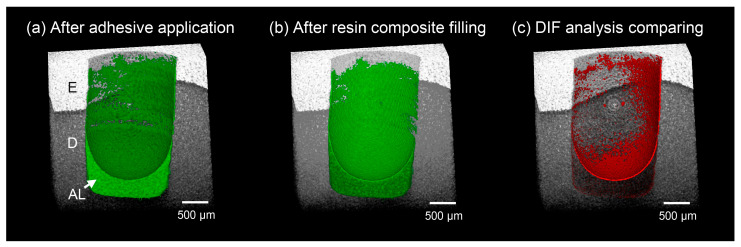
Three-dimensional difference analysis of the SBP adhesive layer before and after resin composite filling. (**a**) Extracted adhesive layer region after adhesive application, (**b**) adhesive layer region after resin composite filling, and (**c**) volume change detected by difference analysis. Green indicates the adhesive layer region, and red indicates regions of volume change. This change was mainly localized at the adhesive–composite interface. E, enamel; D, dentin; AL, adhesive layer; SBP, Scotchbond Universal Plus Adhesive. Scale bar = 500 μm.

Three-dimensional difference analysis between the post-adhesive application and post-filling datasets was performed to evaluate the effect of resin composite filling on adhesive distribution (Figure 6). Although adhesive layer thickness could be measured for all groups using the post-filling dataset (Section 3.3), volume change analysis required reliable binarization of the adhesive in the post-adhesive application dataset. Because SBU, UBQ, and GPB showed grayscale values similar to those of air, reliable segmentation at the adhesive–air interface was not possible. Therefore, quantitative volume change analysis was feasible only for SBP.

For SBP, the total adhesive volume before resin composite filling ranged from 0.507 to 1.197 mm^3^, with a mean of 0.894 ± 0.353 mm^3^. The mean volume change ratio was 6.07 ± 3.29% (Table 4). No statistically significant difference was found between the pre- and post-filling volumes (paired *t*-test: *t* = 2.52, df = 2, *p* = 0.128), although the effect size was large (Cohen’s *d* = 1.45). A decrease in volume was observed consistently in all three specimens. Difference analysis showed that the change was mainly localized to the surface region of the adhesive layer at the adhesive–composite interface. For SBU, UBQ, and GPB, qualitative changes in adhesive distribution were observed after resin composite filling; however, quantitative analysis was limited by insufficient radiopacity.

## 4. Discussion

### 4.1. Radiopacity of Universal Adhesives

The present study demonstrated that among the four universal adhesives evaluated, only SBP exhibited dentin-equivalent radiopacity. Its grayscale value was not significantly different from that of dentin, whereas SBU, UBQ, and GPB showed grayscale values that were statistically indistinguishable from those of air. These findings indicate that most tested universal adhesives have insufficient radiopacity for reliable discrimination from surrounding structures in micro-CT imaging.

This result is consistent with those of previous studies on conventional adhesives. Hotta and Yamamoto reported that 14 of 15 adhesives were less radiopaque than dentin [5]. Oztas et al. found that all eight evaluated adhesives had lower grayscale values than enamel and dentin [6], and de Moraes Porto et al. reported that five of six contemporary adhesive systems were radiolucent relative to dentin [7]. The present study extends these findings to universal adhesives evaluated in a three-dimensional enamel–dentin cavity model using micro-CT.

The dentin-equivalent radiopacity of SBP may be attributed to its radiopaque crosslinking monomer [14,15]. Although both SBP and GPB are HEMA-free, only SBP achieved dentin-equivalent radiopacity, indicating that a HEMA-free formulation alone does not ensure adequate radiopacity. The radiopaque monomer incorporated into SBP appears to be a key differentiating factor, although other physical, mechanical, and bonding properties were beyond the scope of this study. Recent randomized clinical trials have shown that this radiopaque universal adhesive provides clinical performance comparable to that of a conventional universal adhesive over a 2-year period while highlighting its potential to reduce misinterpretation of the radiopaque MDP-based adhesive layer on radiographs [18,19].

The clinical importance of adhesive radiopacity has been previously reported. Kursun et al. showed high false-positive rates for detecting simulated secondary caries adjacent to bonding-agent-lined restorations [8], and Fröhlich et al. reported that multiple SBU layers increased the risk of misdiagnosing radiolucent zones as secondary caries and recommending unnecessary restoration replacement [9]. From a methodological perspective, the near equivalence of the grayscale values of SBU, UBQ, and GPB to those of air limits reliable micro-CT segmentation, thereby restricting quantitative analyses such as interface gap measurement and volume quantification [1].

### 4.2. Intracavity Distribution of the Adhesive Layer

Across all groups, the adhesive layer was consistently thin at the lateral cavity walls and markedly thicker at the cavity floor, with an approximately 3.4-fold difference between the mean lateral wall thickness (66.1 μm) and mean cavity floor thickness (224.2 μm). This pattern is attributable to gravity and air-drying procedures, which displace excess adhesive toward the cavity floor [3]. Similar findings were reported by Sumitani et al. using a two-step self-etch adhesive system in the same cavity model [3], confirming that this pattern is a general characteristic of fluid adhesive applications in enclosed cavity geometries, irrespective of adhesive type.

The clinical implications of adhesive pooling at the cavity floor are significant. Pamir et al. reported that adhesive pooling thickness was significantly associated with false-positive replacement diagnoses [10], and Fröhlich et al. showed that thicker adhesive layers increase the likelihood of radiolucent zone misidentification [9]. For SBU, UBQ, and GPB, the combination of low radiopacity and pronounced floor pooling (up to 321.0 μm for SBU) may increase the risk of diagnostic misinterpretation. In contrast, the dentin-equivalent radiopacity of SBP mitigates this risk regardless of layer thickness. Choi et al. demonstrated that the adhesive layer acts as a stress-absorbing compliance layer during composite polymerization shrinkage [20]; however, this mechanical benefit may be offset by diagnostic challenges when the adhesive is radiolucent.

### 4.3. Regions of Reduced Density in UBQ and GPB

Micro-CT imaging revealed reduced-density regions within the adhesive layers of UBQ and GPB, which became visible only after resin composite filling owing to the radiopaque contrast provided by the resin composite. These findings may reflect material-specific structural irregularities. Because scanning electron microscopy (SEM) or spectroscopic analysis were not performed on the present specimens, the following structural interpretations should be regarded as hypotheses requiring direct experimental confirmation. Yamauchi et al. reported cracks at the GPB–dentin interface using scanning electron microscopy (SEM) and attributed them to the high water content of GPB [21]. In UBQ, the observed microcrack-like features may be related to its HEMA-containing hydrophilic formulation and rapid bonding protocol, which may limit solvent evaporation and promote localized phase separation or stress concentration. The absence of similar findings in SBU suggests that these features are not attributable to HEMA alone. These observations are consistent with reports that one-step universal adhesives form heterogeneous polymer networks prone to phase separation [22] and remain permeable, exhibiting nanoleakage within the adhesive layer [23]; such internal porosity and phase heterogeneity provide a plausible structural basis for the reduced-density regions detected here. Furthermore, the incomplete removal of residual water and organic solvents may exacerbate this internal heterogeneity, contributing to the observed reduction in density [22].

Because UBQ and GPB showed low radiopacity, reliable quantitative defect analysis was not possible, and these findings should be interpreted as qualitative observations only. Further studies using complementary methods, such as SEM and micro-Raman spectroscopy, are required to clarify the nature and clinical relevance of these reduced-density regions. Non-destructive micro-CT detection of such features may complement destructive SEM-based observations [24].

### 4.4. Effect of Resin Composite Filling on Adhesive Distribution

Three-dimensional difference analysis showed that resin composite filling altered the distribution of the adhesive. In the SBP group, the mean volume change ratio was 6.07 ± 3.29%, and the change was mainly localized to the adhesive surface at the adhesive–composite interface. This finding is consistent with previous micro-CT analyses showing that TRI/3D-BON can detect volumetric changes associated with resin composite polymerization [17].

The surface-localized change may be related to the additional polymerization of residual unreacted monomers in the oxygen-inhibited adhesive layer. Sakano et al. reported that the degree of conversion at the adhesive–composite junction did not reach the maximum value even after resin composite light curing, suggesting the presence of an undercured interfacial zone and possible co-polymerization between the adhesive and the overlying composite [25]. Mechanical compression caused by resin composite polymerization shrinkage stress may also have contributed to the observed change [20]. However, the present study could not distinguish among these mechanisms, and further controlled experiments are needed.

For SBU, UBQ, and GPB, reliable quantitative difference analysis was limited because of their low radiopacity. Because their grayscale values were close to those of air, threshold instability at the adhesive–air interface may have produced segmentation artifacts. This finding highlights the methodological advantage of radiopaque adhesives in the micro-CT-based evaluation of adhesive dynamics.

### 4.5. Methodological Advantages and Future Research Directions

The present study extends previous flat-disk radiopacity assessments [5,6,7] by using a cylindrical cavity model prepared in bovine enamel–dentin blocks. This model enabled three-dimensional evaluation of adhesive radiopacity and distribution within an enclosed cavity geometry containing both enamel and dentin surfaces, allowing visualization of adhesive pooling at the cavity floor.

A further methodological advantage was the grayscale-based analytical approach. The adhesive layer region was geometrically defined from sequential micro-CT datasets and analyzed using actual CT grayscale values without threshold-based binarization of the adhesive itself. This approach preserved internal density information and allowed evaluation of adhesive distribution and density variations. In contrast to previous studies on adhesive layer properties, including degree-of-conversion profiling [25] and internal defect observation [21], this approach enables non-destructive, sequential evaluation of the same specimen. Because SBP showed dentin-equivalent radiopacity, it also allowed reliable segmentation and volumetric analysis of the adhesive within the cavity. These findings provide a useful platform for future micro-CT studies evaluating adhesive layer stability under clinically relevant conditions, including different resin composites, curing protocols, thermal cycling, and mechanical loading.

### 4.6. Limitations

This study has several limitations. First, the sample size was small (*n* = 3 per group), which limited the statistical power; therefore, the findings, particularly those related to the SBP volume change analysis, should be interpreted as exploratory. Second, bovine teeth and a standardized cylindrical cavity model were used, which may not fully reproduce the composition, microstructure, or geometry of human tooth cavities. Specifically, bovine dentin has larger and more numerous dentinal tubules and higher permeability than human dentin, which may enhance adhesive infiltration and fluid movement, in turn affecting both the intracavity distribution and measured grayscale values; the caries-free model cavity also lacks the sclerotic or carious substrate and the irregular geometry common in clinical preparations. These differences limit direct extrapolation of the absolute thickness and radiopacity values to clinical practice. Third, the adhesives were evaluated only in the self-etch mode using a single flowable resin composite and a standardized light-curing protocol. To eliminate inter-operator variability, all adhesives were applied by a single, calibrated operator following the respective manufacturers’ instructions. Consequently, operator-dependent variables—such as agitation time, air-drying pressure, application duration, and the number of adhesive coats—were strictly standardized and kept constant rather than being systematically varied; thus, their isolated influences on adhesive distribution and radiopacity remain to be investigated in future studies. In addition, the study was conducted under static conditions without thermal cycling or mechanical loading. Therefore, the present data do not capture the hydrolytic and fatigue-related degradation that progressively alters the adhesive interface in the oral environment and instead reflect the immediate post-curing state rather than long-term clinical performance. Observer blinding was not feasible for the SBP group because of its visually distinguishable radiopacity; however, all measurements were performed by a trained operator according to predefined criteria. Finally, the near equivalence of the grayscale values of SBU, UBQ, and GPB to those of air precluded reliable volume segmentation for these groups, limiting quantitative volume analysis to SBP.

## 5. Conclusions

Within the limitations of this in vitro study, only SBP showed dentin-equivalent radiopacity, whereas SBU, UBQ, and GPB exhibited grayscale values that were statistically indistinguishable from those of air. The adhesive layer was consistently thinner along the lateral cavity walls and thicker at the cavity floor, suggesting the influence of gravity and air-drying procedures. Reduced-density regions were detected in UBQ and GPB only after resin composite filling; however, further investigation is required to determine whether these regions represent structural defects. Three-dimensional volume changes after resin composite filling could be quantified only for SBP, with the changes mainly localized to the adhesive surface region. The cylindrical enamel–dentin cavity model enabled a clinically relevant three-dimensional evaluation beyond conventional flat-disk radiopacity testing. These findings indicate that SBP provides a useful radiopaque adhesive platform for future non-destructive, sequential micro-CT studies of adhesive interface behavior.

## Figures and Tables

**Table 1:** Universal adhesives and resin composites evaluated in this study.

Code	Brand	Main Components	Lot No.	Manufacturer
SBU	Scotchbond Universal Adhesive	MDP, Vitrebond™ Copolymer, HEMA, dimethacrylate resins, filler, ethanol, water, initiators, silane	4897805	Solventum, Maplewood, MN, USA
SBP	Scotchbond Universal Plus Adhesive	MDP, Vitrebond™ Copolymer, dimethacrylate resins, radiopaque cross-linking monomer, filler, ethanol, water, initiators, silane	01746A	Solventum, Maplewood, MN, USA
UBQ	Clearfil Universal Bond Quick ER	Bis-GMA, MDP, HEMA, hydrophilic amide monomer, filler, ethanol, water, NaF, photoinitiators, silane coupling agent	7K0016	Kuraray Noritake Dental, Tokyo, Japan
GPB	G-Premio Bond	MDP, 4-MET, MDTP, BHT, acetone, dimethacrylate resins, initiators, filler, water	2001161	GC, Tokyo, Japan
RC	Clearfil Majesty ES Flow Low	Barium glass, filler, TEGDMA, stabilization agent, photopolymerization catalyst, colorant	620320	Kuraray Noritake Dental, Tokyo, Japan

**Table 2:** Mean 16-bit grayscale values of all tested materials.

Material	Mean ± SD	Group *
Air	33,440 ± 735	a
UBQ	35,471 ± 206	a
SBU	35,662 ± 305	a
GPB	35,851 ± 149	a
SBP	37,716 ± 207	b
Dentin	38,279 ± 187	b
Enamel	40,875 ± 287	c
Resin composite	46,103 ± 164	d

* Different letters indicate statistically significant differences (Games–Howell post hoc test, *p* < 0.05). SBU, Scotchbond Universal Adhesive; SBP, Scotchbond Universal Plus Adhesive; UBQ, Clearfil Universal Bond Quick ER; GPB, G-Premio Bond.

**Table 3:** Adhesive layer thickness (μm) at each measurement position (mean ± SD, *n* = 3).

Position	SBU	SBP	UBQ	GPB
Lateral wall, 0.5 mm depth	35.0 ± 8.2	15.0 ± 17.3	68.3 ± 55.1	48.3 ± 48.0
Lateral wall, 1.0 mm depth	36.7 ± 6.2	25.0 ± 10.0	71.7 ± 33.3	61.7 ± 55.1
Lateral wall, 1.5 mm depth	102.0 ± 97.0	111.7 ± 66.4	93.3 ± 32.5	125.0 ± 25.0
Cavity corner	388.0 ± 195.8	330.0 ± 134.6	302.0 ± 95.6	281.7 ± 69.8
Cavity floor	321.0 ± 209.6	240.0 ± 142.9	194.0 ± 81.6	142.0 ± 108.4

No significant differences in thickness were detected among the four adhesives at any position (one-way ANOVA, *p* > 0.05). SD, standard deviation.

**Table 4:** Total adhesive volume, altered volume after resin composite filling, and volume change ratio (mean ± SD, *n* = 3).

Group	Total Bonding Volume (mm^3^)	Altered Volume (mm^3^)	Volume Change Ratio (%)
SBU	nd	nd	nd
SBP	0.894 ± 0.353	0.060 ± 0.042	6.07 ± 3.29
UBQ	nd	nd	nd
GPB	nd	nd	nd

nd, not determined; volume quantification was not feasible because of noise interference resulting from the near-equivalence of adhesive grayscale values to air, which exceeded the detection limit for reliable segmentation. SD, standard deviation.

## Data Availability

The original contributions presented in this study are included in the article. Further inquiries can be directed to the corresponding author.

## Abbreviations

The following abbreviations are used in this manuscript:

micro-CT	Micro-computed tomography
SBU	Scotchbond Universal Adhesive
SBP	Scotchbond Universal Plus Adhesive
UBQ	Clearfil Universal Bond Quick ER
GPB	G-Premio Bond
SEM	Scanning electron microscopy
RC	Resin composite
